# Supplementary material for: Hair Sample Analysis as a Method of Monitoring Exposure to Bisphenol A in Dogs
Source: Int J Environ Res Public Health. 2022 Apr 11;19(8):4600. doi: 10.3390/ijerph19084600 (PMC9030106; doi:10.3390/ijerph19084600)

Supplementary materials:

Hair Sample Analysis as a Method of Monitoring Exposure to Bisphenol A in Dogs

**Krystyna Makowska [1,*], Julia Martín [2], Andrzej Rychlik [1], Irene Aparicio [2], Juan Luis Santos [2], Esteban Alonso [2]
and Sławomir Gonkowski [3]**

[1] Department of Clinical Diagnostics, Faculty of Veterinary Medicine, University of Warmia and Mazury in Olsztyn, Oczapowskiego 14, 10-957 Olsztyn, Poland; andrzej.rychlik@uwm.edu.pl
[2] Departamento de Química Analítica, Escuela Politécnica Superior, Universidad de Sevilla, C/ Virgen de África, 7, E-41011 Sevilla, Spain; jbueno@us.es (J.M.); iaparicio@us.es (I.A.); jlsantos@us.es (J.L.S.); ealonso@us.es (E.A.)
[3] Department of Clinical Physiology, Faculty of Veterinary Medicine, University of Warmia and Mazury in Olsztyn, Oczapowskiego 13, 10-957 Olsztyn, Poland; slawomir.gonkowski@uwm.edu.pl
* Correspondence: krystyna.makowska@uwm.edu.pl

Table S1 Characterization of dogs included into the study

No.	**Breed**	**Gender**	**Age (years)**	**BCS points**
1	Poodle	female	11	7
2	Mongrel	female	3	7
3	Shih Tzu	female	8	5
4	Golden Retriver	female	8	8
5	German Shepherd	male	9	7
6	Schnauzer	female	7	8
7	Yourkshire Terrier	male	3	3
8	Mongrel	male	4	6
9	Ihasa Apso	female	12	3
10	Shi-tzu	female	3	4
11	Yorkshire Terrier	female	12	6
12	Mongrel	male	1.5	3

13	Poodle	male	2	3
14	Yorkshire Terrier	male	12	9
15	German Shepherd	female	4	7
16	Dalmatian	female	4	3
17	Irish Setter	female	2	4
18	Miniature Poodle	female	5	8
19	Miniature Schnauzer	female	16	7
20	Mongrel	male	14	4
21	Jack Russel Terrier	female	1.5	4
22	Miniature Poodle	female	6	4
23	Mongrel	male	13	5
24	Schnauzer	female	8	4
25	Cocker Spaniel	female	11	8
26	Maltese	female	6	5
27	Bolognese	female	8	9
28	Mongrel	female	3	4
29	Maltese	female	7	5
30	Schnauzer	male	2	4

BCS - body condition score

Method validation

The method was validated according to the International Conference on Harmonization (ICH) guidelines for analytical method validation.

Matrix-matched calibration standards were prepared at eight different analyte concentration levels (from method quantification limits (MQL) to 700 ng/g). The mixtures were vortexed for 2 min and then left to stand for 24 h at 4 ºC in the dark before analysis.

A recovery assay was carried out to validate the accuracy of the method in terms of trueness and precision. Fortified blank samples at three concentrations levels were analyzed. Precision, expressed as relative standard deviation (%, RSD) was determined from triplicate spiked in six different days, and the trueness was evaluated using the recovery data.

Method detection limits (MDLs) and MQLs were calculated from the signal-to-noise ratio (MDLs = 3, MQLs = 10) obtained from injecting several blank samples fortified with decreasing amounts of BPA.

The selectivity of the method was determined by comparing the chromatograms of blank with the corresponding spiked sample.

Table S2. Linear range, MDLs, MQLs, recovery and precision of BPA in fur samples.

Compound	Liner range	MDL	MQL	Recovery (%)			Inter-day precision
	(ng/g)	(ng/g)	(ng/g)	Low level	Medium level	High level	(RSD %)
BPA	4.2-700	1.25	4.20	94	95	99	6

Figure S1. Mass spectrum of BPA.

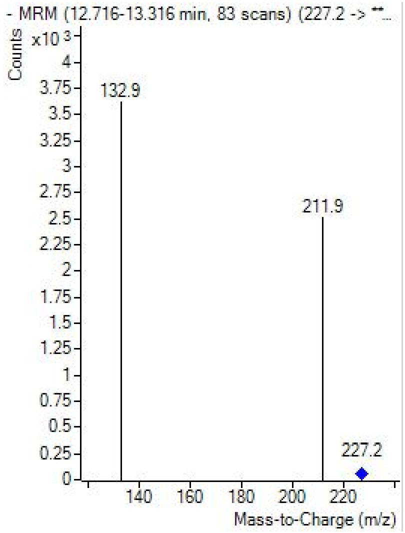

Figure S2. MRM Chromatograms of fur samples spiked at low, medium and high concentration levels.

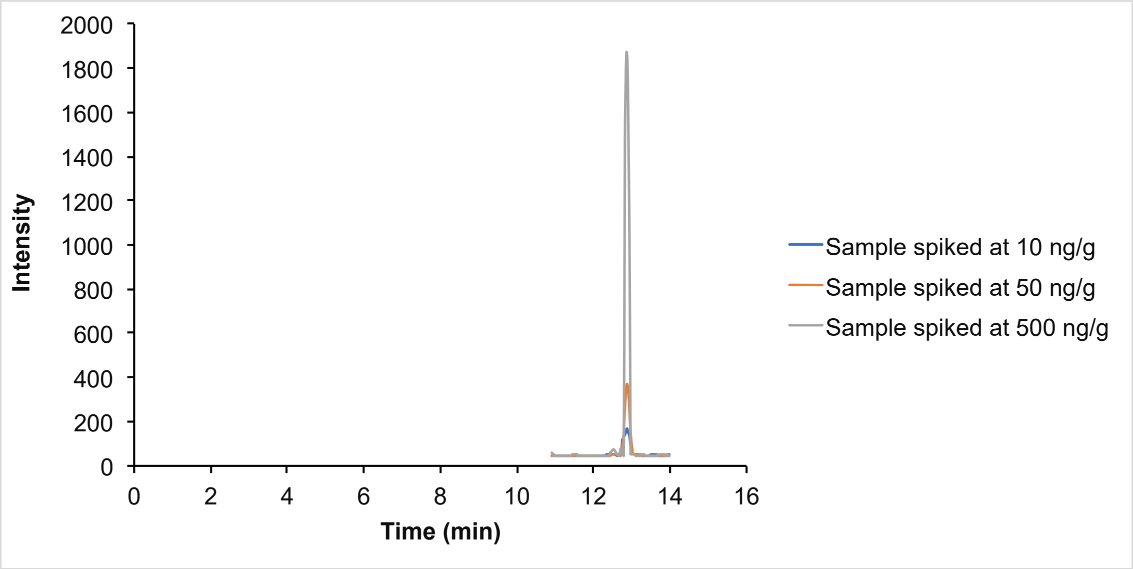

Figure S3. MRM Chromatograms of a real fur sample and a blank matrix.

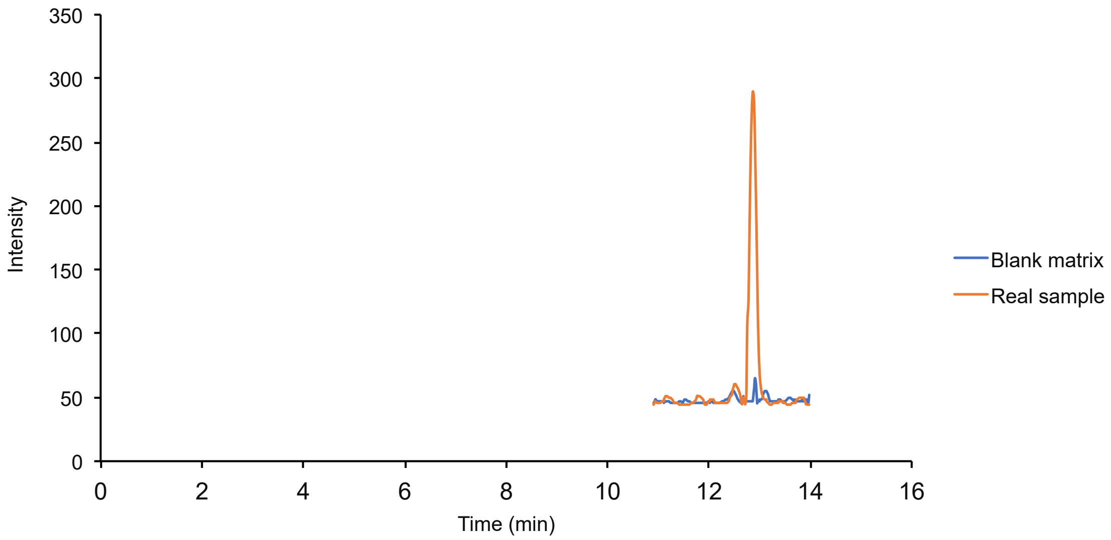

Supplement: Supplementary file 1 [file ijerph-19-04600-s001.zip › ijerph-1619869-supplementary.pdf]
